# High-sensitivity intravascular photoacoustic imaging of lipid–laden plaque with a collinear catheter design

**DOI:** 10.1038/srep25236

**Published:** 2016-04-28

**Authors:** Yingchun Cao, Jie Hui, Ayeeshik Kole, Pu Wang, Qianhuan Yu, Weibiao Chen, Michael Sturek, Ji-Xin Cheng

**Affiliations:** 1 Weldon School of Biomedical Engineering, Purdue University, West Lafayette, IN 47907, USA; 2 Department of Physics and Astronomy, Purdue University, West Lafayette, IN 47907, USA; 3 Department of Cellular & Integrative Physiology, Indiana University School of Medicine, Indianapolis, IN 46202, USA; 4 Key Laboratory of Space Laser Communication and Detection Technology, Shanghai Institute of Optics and Fine Mechanics, Chinese Academy of Sciences, Shanghai 201800, China; 5 Department of Chemistry, Purdue University, West Lafayette, IN 47907, USA

## Abstract

A highly sensitive catheter probe is critical to catheter-based intravascular photoacoustic imaging. Here, we present a photoacoustic catheter probe design on the basis of collinear alignment of the incident optical wave and the photoacoustically generated sound wave within a miniature catheter housing for the first time. Such collinear catheter design with an outer diameter of 1.6 mm provided highly efficient overlap between optical and acoustic waves over an imaging depth of >6 mm in D_2_O medium. Intravascular photoacoustic imaging of lipid-laden atherosclerotic plaque and perivascular fat was demonstrated, where a lab-built 500 Hz optical parametric oscillator outputting nanosecond optical pulses at a wavelength of 1.7 μm was used for overtone excitation of C-H bonds. In addition to intravascular imaging, the presented catheter design will benefit other photoacoustic applications such as needle-based intramuscular imaging.

Cardiovascular disease has been the leading cause of death in the United States and many other developed countries over the past century^1^. Atherosclerosis, a major form of cardiovascular disease, is caused by the chronic accumulation of lipids and fibrous elements within the wall of an artery. This plaque can grow and become clinically symptomatic if it significantly encroaches and obstructs the lumen of the artery. A plaque may also rupture and result in acute coronary syndrome or even sudden death^2^,^3^. Therefore, the early detection of plaques that are vulnerable for rupture is essential in the diagnosis, treatment, and prevention of cardiovascular diseases. Currently, there are no clinically reliable imaging tools to accurately identify vulnerable atherosclerotic plaques, which are characterized post-mortem by a large lipid core and thin fibrous cap^4^. Non-invasive modalities such as X-ray angiography, magnetic resonance, and computed tomography angiography have been used to visualize obstructive stenosis in coronary arteries. However, vulnerable plaques prone to rupture are often non-obstructive or moderately obstructive, thus evading detection by these modalities^5^. Intravascular ultrasound (IVUS) can provide important morphologic information of arteries including lumen geometry, plaque burden, and vessel structure. However, the sensitivity and specificity for differentiation of plaque composition is limited, partly due to the lack of chemical contrast with IVUS^6^,^7^. Intravascular optical coherence tomography^8^,^9^ have been reported, but these two optical imaging modalities fail to provide necessary imaging depth and chemical specificity for vulnerable plaque detection. Near infrared spectroscopy^10^ provide chemical selectivity but it lacks the spatial resolution to define the lipid core size and its detection sensitivity is compromised by scattered photons.

Catheter-based intravascular photoacoustic (IVPA) imaging^11^,^12^,^13^,^14^, on the basis of converting the overtone vibrational absorption in an arterial tissue into thermoelastic waves detectable with an ultrasonic transducer^15^,^16^,^17^, is an emerging modality with potential of bridging the aforementioned gaps. IVPA imaging offers the following advantages. First, the optical absorption-induced contrast provides a unique approach to differentiate chemical composition of arteries. Second, the imaging depth of IVPA is extended beyond the ballistic regime owing to the diffused photon absorption and 2–3 orders of magnitude lower acoustic scattering in tissues compared to optical scattering^18^. Third, by sharing the same detector, IVUS is inherently compatible with IVPA imaging. Such a hybrid modality provides complementary information of the tissue.

A clinically feasible IVPA catheter should be of small diameter, flexible, capable of imaging through blood, and acquiring images with high sensitivity and chemical specificity at an acceptable frame rate. These requirements collectively render the design and fabrication of a high-performance IVPA probe to be one of the most challenging tasks in the photoacoustic imaging field. A number of groups have reported IVPA catheters with diameter approaching the clinical requirement of about 1 mm^11^,^12^,^13^,^14^,^19^,^20^,^21^,^22^,^23^,^24^. Specifically, the Emelianov group reported two designs of IVPA catheters, one based on side fire fiber and the other based on mirror reflection^11^. Both designs were based on a front-to-back arrangement of the light delivery element and ultrasonic transducer. The Chen group introduced another design of IVPA catheter based on parallel arrangement of side-firing fiber and transducer, where two different frequencies, 35 MHz and 80 MHz, of the transducer were performed to demonstrate an outstanding axial resolution of 35 μm^22^. The Xing group introduced an intravascular confocal photoacoustic probe with dual-element ultrasound transducer^20^. The Song group reduced the diameter of IVPA catheter probe to 1.1 mm by carefully arranging the positions of the optical and acoustic elements^19^. In their most recent work, the authors further reduced the probe diameter to 0.9 mm^21^. Despite these advances, sufficient arterial imaging depth has not been shown for these single-element transducer based IVPA catheters, largely because the optical and ultrasonic waves were cross overlapped in a very limited space. Although the overlap range can be altered by changing the coupling angle^25^, it is hard to maintain the photoacoustic sensitivity constant along the millimeter-scale imaging depth. Furthermore, the IVUS and IVPA images in these non-collinear designs are not truly co-registered along the imaging depth, which may lead to poor localization of artery and plaque features. Assembly of the non-collinear design is also nontrivial, as all the components must be constrained to a limited space. To maximize the overlap of incident optical field and generated acoustic wave, our team recently demonstrated a coaxial design based on a ring-shaped transducer^26^. However, the outer diameter of the probe (2.9 mm) needed to be further reduced for clinical compatibility.

Here, we report an IVPA catheter probe based on a collinear alignment of optical and acoustic waves to overcome the drawbacks in aforementioned IVPA catheters. In our approach, an optical beam delivered through a 365-μm-core multimode fiber (MMF) with a low numerical aperture of 0.22 allowed quasi-uniform illumination along the imaging depth. An outer diameter of 1.6 mm was reached for the catheter tip through careful arrangement of the optical and acoustic elements. This collinear design ensured an efficient overlap between optical and photoacoustic waves over 6 mm imaging depth. The capability of our collinear design catheter probe was evaluated through *ex vivo* high-speed IVPA imaging of a diseased porcine carotid artery and a human coronary artery, with optical excitation via a lab-built optical parametric oscillator (OPO) outputting optical pulses at 1.7 μm wavelength and 500 Hz repetition rate.

## Results

### Collinear IVPA catheter

**Figure 1:**
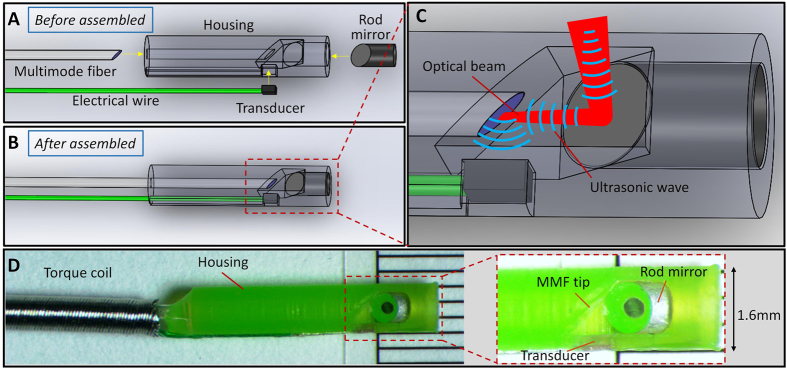
Collinear IVPA catheter probe. (**A**) Main components of the collinear catheter before assembly. (**B**) Assembled IVPA catheter probe. (**C**) Zoom-in view of the catheter tip with illustration of collinear overlap between optical and ultrasonic waves. (**D**) Photograph of the fabricated catheter probe with a diameter of 1.6 mm, and the detailed structure of the catheter tip (inset).

The design of the collinear catheter probe is shown in Fig. 1. A section of MMF is used for delivery of light to the probe. The distal end of the MMF is polished to 45° for ultrasonic wave reflection, while the optical wave still propagates forward after the polished end when the optical fiber is submerged in an aqueous environment. A single-element ultrasonic transducer is placed parallel to the MMF, with its sensing area facing the polished fiber plane. Therefore, the optical and ultrasonic paths are collinear after encountering the polished surface (shown in Fig. 1C). A 45° rod mirror right after the delivery fiber is served to redirect both the optical and ultrasonic waves perpendicularly for side-view illumination and imaging. It should be noted that the ultrasound trace after the rod mirror is designed to be perpendicular to its receiving plane within the catheter housing to prevent direct ultrasound wave venting from the transducer as shown in Fig. 1C, which may cause errant image reconstruction. This design ensures that optical and acoustic waves are collinear within a large tissue depth. The components involved are installed in a well-designed housing with an outer diameter reasonably compatible in clinical settings, thus greatly simplifying the catheter assembly process. A photograph of the lab-fabricated catheter probe is shown in Fig. 1D, with its outer diameter measured to be 1.6 mm. The detailed procedure of the catheter fabrication can be found in the Methods section.

### Architecture of the IVPA imaging system

**Figure 2:**
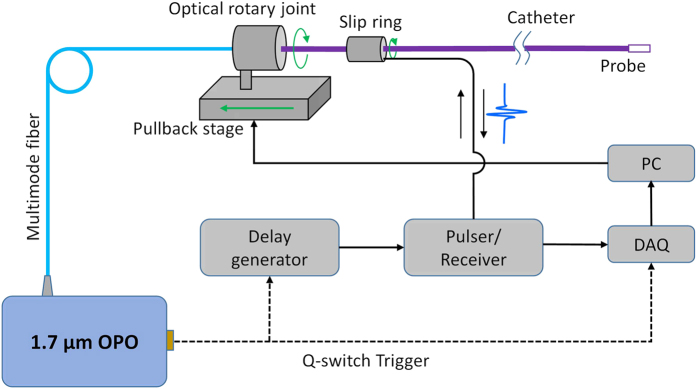
Architecture of the IVPA imaging system. OPO, optical parametric oscillator; DAQ, data acquisition; PC, personal computer.

The schematic IVPA imaging system is shown in Fig. 2. A lab-built potassium titanyl phosphate (KTP)-based OPO emitting at 1.7 μm with a repetition rate of 500 Hz and pulse width of ~13 ns was used as the optical excitation source for photoacoustic imaging^27^. Light is coupled to the catheter via a MMF and an optical rotary joint. The pulse energy on the catheter tip was controlled to be ~120 μJ, corresponding to an energy density of ~30 mJ/cm^2^ at the tissue surface, which is below the 1.0 J/cm^2^ ANSI safety standard for skin at 1.7 μm^28^. 3-D imaging of the system was enabled by a rotational scanning system and a pullback stage. Sequential photoacoustic and ultrasound signals were generated and detected with a proper time delay. The detailed information of the scanning system and data acquisition can be found in Methods section. The current imaging speed of our system is 1 frame per second, which is 50 times faster than traditional IVPA imaging systems based on 10-Hz Nd:YAG lasers^11^,^12^,^14^,^22^. The collinear catheter based imaging system was subsequently characterized for performance evaluation and validated with *ex vivo* artery imaging.

### Characteristics of spatial resolution and imaging depth

**Figure 3:**
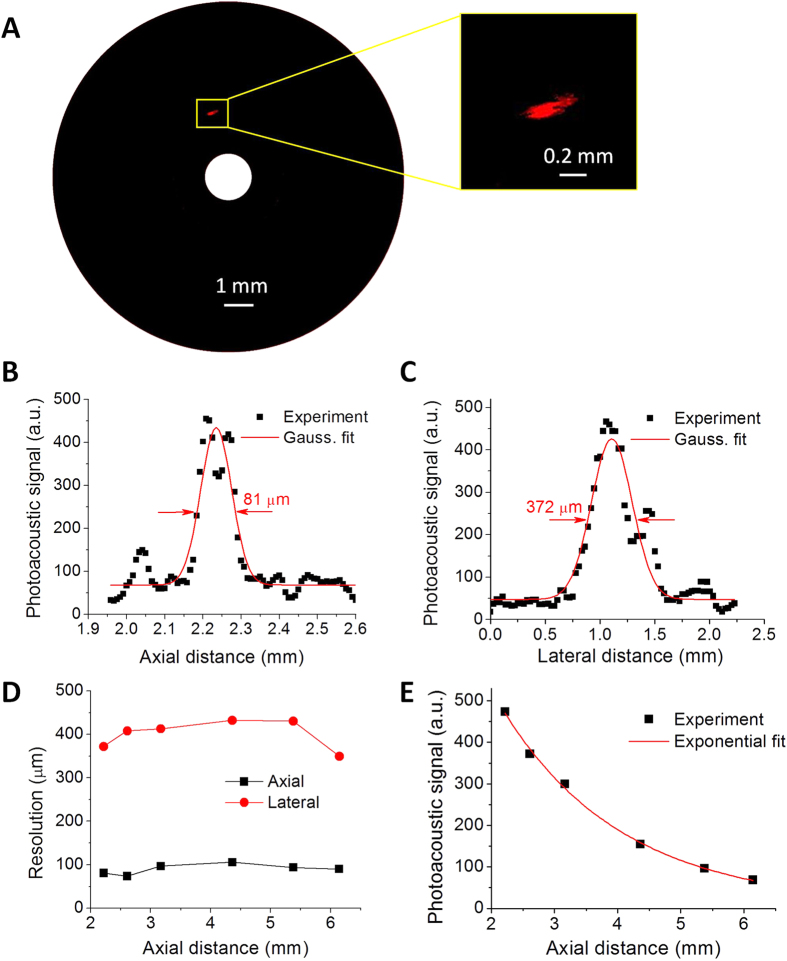
Characterization of spatial resolutions and imaging depth. (**A**) Cross-sectional photoacoustic image of a single 7-μm carbon fiber. (**B**) Axial resolution and (**C**) lateral resolution obtained at an axial distance of 2.2 mm. (**D**) Axial and lateral resolutions at different axial distances. (**E**) Magnitude of photoacoustic signal produced by the single carbon fiber at different axial distances.

The spatial resolution of our catheter was evaluated by photoacoustically imaging a carbon fiber with 7-μm diameter. The carbon fiber serves as a perfect target to determine the spatial resolution of IVPA imaging due to its strong optical absorption and well-defined thin diameter. The carbon fiber was positioned parallel to the catheter probe with a variable distance controlled by a translation stage. The experiments were performed in deuterium oxide (D_2_O) medium because of its lower optical absorption at 1.7 μm compared to water^29^. Figure 3A shows the reconstructed cross-sectional photoacoustic image of carbon fiber with a rotational catheter scanning. The inset shows the zoom-in view of the carbon fiber image. The generated photoacoustic signals along the axial and lateral directions centered at the carbon fiber position are plotted in Fig. 3B,C to determine the spatial resolution. The axial and lateral resolutions are derived from the full width at half maximum of Gaussian fit of these results. An axial resolution of 81 μm and lateral resolution of 372 μm were obtained at a radial distance of 2.2 mm. Spatial resolutions for photoacoustic imaging at different axial distances were obtained similarly by changing the position of the carbon fiber as displayed in Fig. 3D. The axial resolutions are found to fluctuate around 80 μm, which are primarily determined by the bandwidth of the transducer, while lateral resolutions are found to varying from 350 μm to 430 μm, which may be due to the non-focus property of ultrasonic transducer. The magnitude of the photoacoustic signals at different axial distances is plotted as well in Fig. 3E. It shows approximately an exponential decay along the axial direction. Notably, the overlap range between optical beam and ultrasonic wave is found to be over 6 mm, which has not been achieved for non-collinear catheter designs previously reported. This imaging depth is sufficient for intravascular applications.

### Chemical specificity validation with a lipid-mimicking phantom

**Figure 4:**
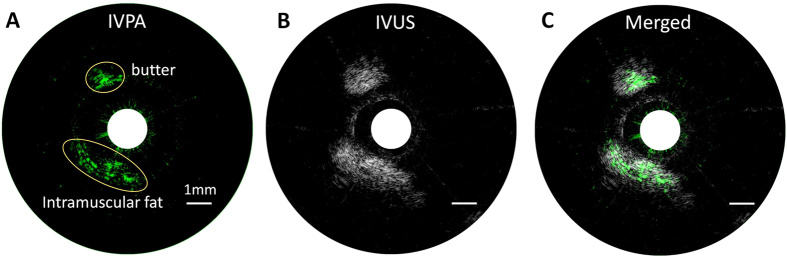
IVPA imaging of a lipid-mimicking phantom comprised of a butter rod and a piece of porcine intramuscular fat. (**A**) IVPA image in green color map. (**B**) IVUS image in gray color map. (**C**) Merged photoacoustic and ultrasound image. The 1 mm scale bar applies to all panels. The shapes and positions of butter and fat are highlighted in yellow ellipses in panel (**A**).

A lipid-mimicking phantom composed of a butter rod and a portion of porcine intramuscular fat were employed for photoacoustic imaging to evaluate the sensitivity and validate the chemical specificity of our system. Similar to pathologic lipid deposition in atherosclerosis, both butter and intramuscular fat are abundant in CH_2_ groups, which exhibit strong absorptions at their first overtone transitions around 1.7 μm. Porcine intramuscular fat serves as a reliable model of pathologic lipid deposition, thus validating the feasibility of our photoacoustic catheter probe to perform intravascular imaging. The procedure of phantom preparation can be found in Methods section. Both photoacoustic and ultrasound images of the phantom were shown in Fig. 4. We observed that both butter and fat can be identified from both photoacoustic and ultrasound images, with strong association between them on position and morphology. The signal-to-noise ratios for butter and fat in photoacoustic image were calculated to be 38 and 18, respectively, while the signal-to-noise ratios are 30 and 46 for butter and fat in ultrasound mode. The photoacoustic signals are specific for the density of CH_2_ bond in these two targets, while the ultrasound signals are related to the overall structural properties. These results from the lipid-mimicking phantom validate the performance of photoacoustic and ultrasonic imaging of lipid with our catheter, indicating our imaging system can be used for reliable IVPA and IVUS imaging of an artery.

### IVPA imaging of lipid-laden carotid artery excised from Ossabaw swine

**Figure 5:**
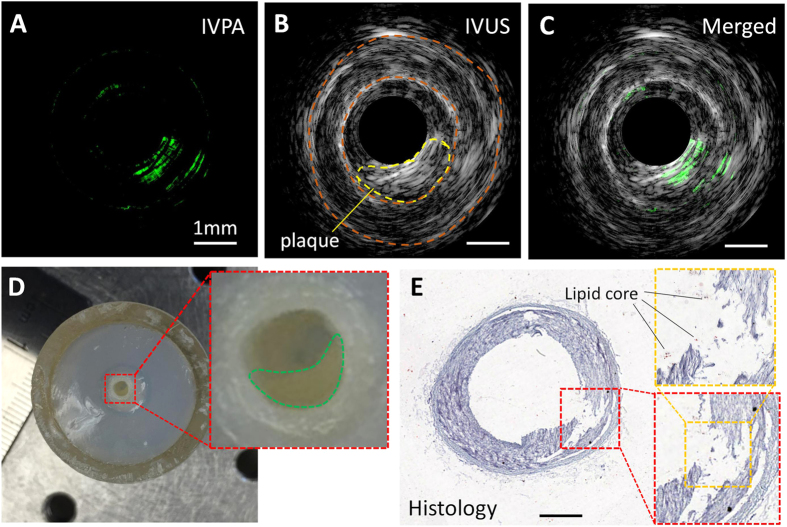
Co-registered IVPA/IVUS imaging of a swine carotid artery *ex vivo*. (**A**) IVPA image. (**B**) IVUS image. (**C**) Merged IVPA/IVUS image. (**D**) Gross photograph of the artery segment with obstruction traced in the inset. Scale is shown by the ruler beside. (**E**) Histology of the cross section of the artery at the IVPA imaging position. The lipid deposition is suggested by the blank area highlighted in the box defined by yellow dashed lines. The 1 mm scale bar applies to all panels except (**D**).

The performance of our IVPA imaging system was validated by *ex vivo* imaging of a diseased carotid artery. A segment of the artery with suspected plaque (shown as artery stenosis in Fig. 5D) was selected as the imaging target. Co-registered IVPA/IVUS images were obtained as shown in Fig. 5A–C. From the IVUS image in Fig. 5B, the characteristic three-layer appearance and luminal area of the carotid artery can be visualized, with the suspected plaque region and inner and outer boundaries of the artery inscribed, which agree well with gross inspection at the plaque position (Fig. 5D). Strong photoacoustic signal within the plaque region shown in Fig. 5A indicates a possible lipid-rich core of the plaque. The co-registered image in Fig. 5C shows the overlap between the photoacoustic and ultrasonic signal at the plaque area. The imaged cross-sectional region was further sectioned and stained for histology, as shown in Fig. 5E. The lumen size and arterial structure were verified by the histology. The plaque position was highlighted by a red box. The lipid deposition, which might have been leached out during the histology process, is suggested by the blank area. Some debris of the lipid core can still be visualized in the zoom-in view indicated by black lines. The torn region of the artery in Fig. 5E is most likely an artifact due to improper cryosectioning during histology.

### IVPA imaging of fresh coronary artery excised from human patient

**Figure 6:**
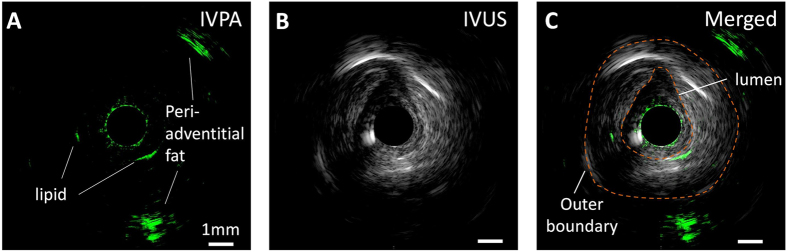
IVPA/IVUS imaging of a perfused fresh human right coronary artery dissected from an explanted heart. (**A**) IVPA image. (**B**) IVUS image. (**C**) Merged IVPA/IVUS image. The 1 mm scale bar applies to all panels.

We further validated the capability of our collinear catheter design by *ex vivo* imaging a perfused fresh right coronary artery from a human patient. The artery segment was imaged with a 3-D scanning system composed by an optical rotary joint and a linear pullback stage. At a particular longitudinal position, we identified a region of interest with strong photoacoustic signal in the arterial wall, which could possibly indicate lipid depositions as shown in Fig. 6. Furthermore, we observed intense photoacoustic signal peripheral from the vessel wall with an imaging depth of 4.3 mm, suggested that our collinear IVPA imaging system is able to penetrate through the entire arterial wall to reach the surrounding perivascular fat that was retained on the excised vessel.

## Discussion

Compared to conventional non-collinear catheter designs, where optical and ultrasonic waves only overlap partially within a limited range^11^,^12^,^14^,^19^,^20^, the biggest advantage of our collinear catheter design is that the generated ultrasonic wave shares the same path with the optical excitation beam, which provides a highly efficient overlap between optics and acoustics along the entire imaging depth. This development results in optimal photoacoustic sensitivity over an imaging depth over 6 mm experimentally, allowing reliable access of the deeper component information in the entire arterial wall, including perivascular fat. Even so, the photoacoustic signal along an A-line still decays exponentially as shown in Fig. 3E. This decay can originate from a number of reasons including optical beam divergence, optical absorption/scattering in imaging environment, the reduced collection angle of photoacoustic wave at an increased depth due to its divergent property and the unfocused ultrasound transducer, and acoustic loss in medium. Some approaches to reduce signal decay include integrating a gradient-index lens in the catheter to improve the optical beam focusing, introducing external wavefront shaping method to focus the light beam deeper inside the tissue^30^, and using a quasi-focused transducer to enhance the acoustic receiving efficiency.

The diameter of our current fabricated catheter probe is 1.6 mm, mainly limited by the size of the rod mirror (1 mm diameter). With a reduced diameter of 0.5 mm for the rod mirror, the catheter probe can be further reduced to ~1 mm in diameter, which is similar to the size of commercially available IVUS catheter probes^31^.

The imaging speed of our current system is 1 frame per second, which is based on the 500 Hz repetition rate OPO and one revolution per second rotation speed of the catheter. Considering the lateral resolution of ~425 μm at an axial distance of 5 mm, the number of A-lines for each cross-sectional image can be reduced to 75, which would allow a maximum imaging speed over 6 frames per second. We also intend to develop a similar laser system at a higher repetition rate of 2 kHz, which will further improve our imaging speed to approach that of commercial *in vivo* intravascular imaging systems^31^.

In conclusion, we demonstrated a miniature IVPA catheter probe with collinear overlap between the optical and acoustic fields. This design enabled high-quality IVPA imaging of the entire artery wall from lumen to perivascular fat. The lab-fabricated collinear catheter was evaluated for spatial resolution characterization with a 7-μm carbon fiber and chemical composition validation by using a lipid-mimicking phantom. The axial and lateral resolutions were found to be around 80 μm and 400 μm, respectively, over an imaging depth larger than 6 mm. With a co-registered IVPA/IVUS imaging system based on a lab-built 500 Hz OPO at 1.7 μm, the catheter was used to image a diseased carotid artery and a human coronary artery *ex vivo*, resulting in IVPA/IVUS images showing a lipid-rich plaque that corresponds with gross inspection. These results collectively help the photoacoustic imaging community to move towards the realization of *in vivo* IVPA imaging in the clinic.

## Methods

### Ethics statement

All the experiment protocols in this study were approved by the Institutional Biosafety Committee of Purdue University, and in accordance with the approved guidelines. The experiments involving human coronary artery were approved by Human Research Protection Program of Purdue University, and the informed consent was obtained from all subjects.

### Fabrication of a collinear catheter

The collinear IVPA catheter was fabricated according to the following procedures: (1) a catheter housing with structure shown in Fig. 1A was 3-D printed with micro-resolution stereolithography process (Proto Labs, Inc.); (2) a custom-designed single-element transducer with dimensions of 0.5 × 0.6 × 0.2 mm^3^, center frequency of 42 MHz and bandwidth of 60% (Blatek, Inc.) was fitted in the square hole, with its sensing area facing the reflection plane; (3) a section of MMF with core/cladding diameter of 365/400 μm, NA of 0.22 (FG365LEC, Thorlabs, Inc.) was polished to 45° at one end and 90° at the other end with a fiber polisher (NANOpol, ULTRA TEC Manufacturing, Inc.). The 45°-polished fiber end was then inserted into the housing until matching with the acoustic reflection plane. The MMF was rotated precisely to ensure the collinearity between the optical and acoustic waves; (4) a rod mirror with diameter of 1 mm (Edmund Optics, Inc.) was inserted into the distal end of the catheter housing, and tuned to ensure the optical beam illuminate radially; (5) the components were glued, a torque coil was employed to enclose the optical fiber and electrical wire of the transducer, and a fiber connector was installed on the distal end of the catheter. The relative positions among each components are optimized by monitoring the photoacoustic signal in real time under aqueous environment.

### Scanning system and data acquisition

An optical rotary joint together with a slip ring were used to control the rotational scanning of the catheter (Fig. 2). An additional pullback stage installed with the rotary joint was used to enable 3-D imaging. The trigger signal provided by the *Q*-switch of OPO was used to synchronize the data acquisition of IVPA and IVUS signals. A time delay of 10 μs was applied to ultrasound pulser via a delay generator (37000-424, Datapulse, Inc.). Both IVPA and IVUS signals are sequentially detected by the same transducer installed in the catheter and received by a pulser/receiver (5073 PR, Olympus, Inc.) with an amplification factor of 39 dB. A data acquisition (DAQ) card (ATS9462 PCI express digitizer, AlazerTech, Canada) with 16-bit digitization and 180 MS/s sampling rate was used to digitize and transfer the generated signals via a LabView software.

### Lipid-mimicking phantom preparation

The 2.5% agarose gel made from agar powder and D_2_O approximately mimics the tissue environment. A butter rod with a diameter of ~1.5 mm and a small piece of intramuscular fat were embedded in the agarose gel as imaging targets. A central hole in the phantom was reserved for catheter insertion. The phantom was fully submerged in D_2_O during imaging experiment to ensure a lower optical loss at 1.7 μm.

### Carotid artery specimen

The atherosclerotic carotid artery was harvested from a miniature Ossabaw swine, which was fed with high-fat/cholesterol/fructose diet, and then fixed in 10% formalin. Before imaging experiment, a segment of artery with suspected plaque was selected and cut as a region of interest with the aid of a microscope. The artery segment was then held by agarose gel and submerged under D_2_O for imaging experiment.

### Human coronary artery sample

The right coronary artery was harvested from an explanted human heart at the time of transplant. We excised the vessel from the ostium to 6 cm distally, leaving approximately 5 mm of surrounding perivascular fat attached. The ostium was cannulated with an 8 F introducer sheath and side branches were ligated to allow for pressure perfusion. The artery was then pinned in a Sylgard® 184 Silicone Elastomer tray and submerged in phosphate-buffered saline at room temperature and was perfused to mimic physiologic pressure during imaging.

## Additional Information

**How to cite this article**: Cao, Y. *et al.* High-sensitivity intravascular photoacoustic imaging of lipid–laden plaque with a collinear catheter design. *Sci. Rep.* **6**, 25236; doi: 10.1038/srep25236 (2016).
